# Polymers as Encapsulating Agents and Delivery Vehicles of Enzymes

**DOI:** 10.3390/polym13234061

**Published:** 2021-11-23

**Authors:** Adejanildo da S. Pereira, Camila P. L. Souza, Lidiane Moraes, Gizele C. Fontes-Sant’Ana, Priscilla F. F. Amaral

**Affiliations:** 1 Escola de Química, Universidade Federal do Rio de Janeiro, Rio de Janeiro 21941-909, Brazil; adejanildosp@gmail.com (A.d.S.P.); camila_lins@hotmail.com.br (C.P.L.S.); lidiane@eq.ufrj.br (L.M.); 2 Biochemical Processes Technology Department, Chemistry Institute, Universidade do Estado do Rio de Janeiro, Rio de Janeiro 20550-013, Brazil; gizele.santana@uerj.br

**Keywords:** polymers, encapsulation, enzymes, chitosan, sodium alginate

## Abstract

Enzymes are versatile biomolecules with broad applications. Since they are biological molecules, they can be easily destabilized when placed in adverse environmental conditions, such as variations in temperature, pH, or ionic strength. In this sense, the use of protective structures, as polymeric capsules, has been an excellent approach to maintain the catalytic stability of enzymes during their application. Thus, in this review, we report the use of polymeric materials as enzyme encapsulation agents, recent technological developments related to this subject, and characterization methodologies and possible applications of the formed bioactive structures. Our search detected that the most explored methods for enzyme encapsulation are ionotropic gelation, spray drying, freeze-drying, nanoprecipitation, and electrospinning. α-chymotrypsin, lysozyme, and β-galactosidase were the most used enzymes in encapsulations, with chitosan and sodium alginate being the main polymers. Furthermore, most studies reported high encapsulation efficiency, enzyme activity maintenance, and stability improvement at pH, temperature, and storage. Therefore, the information presented here shows a direction for the development of encapsulation systems capable of stabilizing different enzymes and obtaining better performance during application.

## 1. Introduction

Enzymes are valuable molecules for several reasons, including mild reaction conditions, biodegradability, selectivity, high yields, and renewability. In this sense, industries are increasingly demanding their use in quite a few products and processes, mainly in the textile, detergent, starch, pharmaceutical, and fuel sectors [1].

The use of agro-industrial wastes [2,3], media optimization [4], and many modern techniques, such as protein engineering [5] and directed evolution [6], has managed to reduce enzyme production costs and provide many interesting new applications. Even so, the immobilization of these biocatalysts is essential for industrial use because of operational stability and reusability [7]. Besides, immobilization is also a crucial technique for the controlled release of these catalytic proteins at specific locations (target site), at a specific rate, or in response to environmental triggers, such as pH, ionic strength, temperature, or enzymatic activity [8]. These features are important for pharmaceutical applications and can improve the technical–functional performance of these molecules [9].

Numerous immobilization techniques have been studied and are still under investigation to obtain robust high activity biocatalysts, and they are divided into three categories: adsorption on a carrier (support), encapsulation in a carrier (entrapment), and crosslinking (carrier-free) [10]. Regardless of the immobilization strategy, polymers certainly play a crucial role in this process: the use of synthetic, natural, inorganic, and smart polymers has been reported so far [10].

The entrapment of an enzyme in a polymer network allows substrates and products to pass through while retaining the enzyme; it can also be designed to release the enzyme in a controlled manner at the target site under specific conditions [11]. Usually, the enzyme does not interact with the polymer, reducing problems of active site block which are faced by adsorption techniques frequently used for industrial applications [12]. However, for these uses, a disadvantage would be that physical restrictions do not prevent enzyme leakage. Hence, some strategies to overcome this problem have been proposed, such as the addition of a second polymer [13]. For controlled release, this characteristic is an advantage used for enzyme delivery [14]. In the present review, we will focus on entrapment techniques used to encapsulate enzymes using polymers.

Several enzyme entrapment methods are reported, and they generally involve mixing the enzyme with a monomer solution, followed by polymerization [15]. Ionic gelation [13], spray drying [16], freeze-drying [17], nanoprecipitation [18], and electrospinning [19] are the latest, most used, and efficient methods to encapsulate enzymes.

The most important parameters to evaluate enzyme immobilization are immobilization yield, global enzyme activity yield, expressed activity (recovered activity or activity recovery), and enzyme loading [20]. Physical characterization of those biocatalysts generally involves particle size and density determination, hydrophobicity measurements, and mechanical robustness evaluation [10]. All these parameters and the physical characterization related to the encapsulation of enzymes by polymers will be addressed in the present review.

Despite the numerous reviews related to enzyme immobilization found in the literature [7,10,11,12], none of them focus on the encapsulation procedures of these molecules. Therefore, in the present review, we aimed at describing polymers as encapsulating agents for enzymes, the recent technological developments related to this subject, and depicting characterization methodologies and possible applications of these bioactive materials.

## 2. Polymeric Materials

Polymers are large molecules consisting of multiple repetitions of one or more units, known as monomers, which react to form polymer chains by the process of polymerization and can present peculiar properties [21]. These large molecules can be classified into three categories according to their source: natural, synthetic, and semi-synthetic, and all of them can be used as encapsulating material for a variety of biomolecules. An ideal material for encapsulation requires good rheological properties, stabilization, non-reactivity with the core material, solubility in non-toxic solvents, non-hygroscopicity, and the possibility of releasing the core material under specific conditions [22].

Natural polymers, or biopolymers, are synthesized from plants, animals, and microorganisms and are mainly composed of proteins (gelatin, collagen, and albumin), polysaccharides (starch, cellulose, agarose, and chitosan), and lipids (paraffin, beeswax, and stearic acid). Biopolymers are largely used in the food and medicine industries due to their non-toxic character, biodegradability, and biocompatibility [23,24].

Although biopolymers generally display unchangeable properties, which may hinder their interaction with various substances, these materials offer a large diversity as matrices and are biodegradable, producing non-toxic by-products. In contrast, they can generate immunogenic reactions and present an uncontrollable rate of degradation [25].

Synthetic polymers are laboratory synthesized molecules, which mainly include polyamide, polyethylene glycol, poly (vinyl acetate), and polyethylene. This type of polymer exhibits easily tailored properties, which enables their adaptation to several applications, and they also display an inert behavior under physiological conditions. Moreover, synthetic polymers present mechanical and chemical stability and good reproducibility [26].

Tunable properties, endless forms, and established structures are some advantages of synthetic polymers over natural ones. On the other hand, some synthetic substances can be harmful to human beings, and are thus avoided by the food and drug industries [27].

The semi-synthetic polymer is a combination of a synthetic and a natural substance, or a blend, enhancing the best qualities and diminishing the undesirable characteristics of each material [28].

Considering the polymer chain’s backbone type, polymers can also be divided into organic polymers—carbon core—and inorganic polymers, constituted by elements other than carbon. Inorganic polymers are amorphous substances formed by a chain of SiO_4_ and AlO_4_ tetrahedra, which share all oxygen atoms. This type of polymer displays good physical and chemical properties, such as high compressive strength, thermal stability, and low permeability [29]. In contrast, organic polymers exhibit a close analogy to the environment predominant in homogeneous polymerization and do not require complex pathways to be produced. Organic–inorganic polymer hybrids can also achieve unique properties once organic compounds are frequently dispersed in an inorganic matrix at a molecular level [30].

Another type of polymeric material is the smart polymer, a stimulus-responsive polymer network that changes its conformation dramatically when slight variations occur in the medium, such as in temperature, pH, or ionic strength [10]. Smart polymers undergo large reversible, physical, or chemical changes and can reverse the transitions. Furthermore, smart polymers can be biocompatible, even though they can be hard to handle and sterilize [31].

Polymers are applied mainly for biomolecule encapsulation and, depending on the chosen material, variable properties can be obtained. Some substances can be more suitable for encapsulating certain molecules than others, and costs, extrinsic and intrinsic factors, and objectives must be considered in this choice. Polysaccharides, vegetable and animal proteins, and gums are common natural encapsulating materials, but synthetic polymers are also used to encapsulate active ingredients [10].

## 3. Most Used Polymers for Enzyme Encapsulation

### 3.1. Chitosan

Chitosan is a natural polycationic linear polysaccharide ((1 → 4)-β-linked 2-amino-2- deoxy-d-glucose) derived from chitin. Chitin is an abundant renewable polymer, and chitosan, its deacetylated derivative, is a widely used biopolymer [32]. Shrimps, crabs, and other crustaceans’ shells have chitin (β-(1 → 4-*N*-acetyl-d-glucosamine) in their composition, which can react with alkaline sodium hydroxide, leading to an *N*-deacetylation product, also called chitosan [33].

Electrostatic interactions with molecules containing negatively charged groups are developed as the amino groups of chitosan and its protonation, which generates NH^3+^, together with its linear chains, react. Furthermore, the presence of this functional group and a hydroxyl on chitosan chains allows for chemical modifications [34].

Chitosan has many advantages for enzyme immobilization, such as biocompatibility, non-toxicity, low allergenicity, and biodegradability. Moreover, antioxidant, antimicrobial, and antitumor activities have been reported for this molecule, and these properties are affected by its degree of deacetylation and its molecular weight [35]. Therefore, chitosan is an important shell material to entrap enzymes that can be used for several applications in different fields. As enzyme immobilization matrices, chitosan-based materials are used primarily in the form of gels of different geometrical configurations, such as beads/spheres, membranes, fibers, and sponges [36].

Different studies have proposed strategies to promote the chitosan transition into sol-gel in recent years. Chitosan gels can essentially be formed in two ways: by “physical” or “chemical” reticulation processes. In the latter case, permanent networks are formed when covalent bonds among chains are exploited. Typically, glutaraldehyde, diglycidyl ether, diacrylate, diisocyanate, or small molecules are used in the preparation of chemical gels of chitosan. However, it is a concern that the toxicity of the majority of such molecules could represent a risk to the environment and living beings [37].

Due to its lower toxicity, physical chitosan gels are, perhaps, more suitable for use as systems for enzyme entrapment. Moreover, the possibility of tuning extent and the rate of gel swelling, mechanical behavior, and degradation are also appealing characteristics of physical chitosan gels. By varying physical and chemical parameters—temperature, ionic strength, and pH—or adding proper counterions, the physical gels are likely to transition to sol-gel. Entangled networks are formed when hydrophobic interactions and electrostatic and hydrogen bonds react. Ionic strength and pH, in relation to ionic interactions, are crucial in gel formation [38].

Chitosan concentration exerts a strong influence on gel formation for enzyme retention by the capsules/beads. According to Nunes et al. [3], when the chitosan concentration was increased from 2 to 5% (*w/v*), the enzyme encapsulating efficiency increased significantly.

Some biopolymers, such as chitosan, alginate, and carrageenan, can be used in the preparation of hybrid supports in order to entrap enzymes. Chitosan can play a key role in the preparation of hybrid polymers, leading to polyelectrolyte complex products formation when in the presence of natural polyanions. Chitosan–alginate hybrid gel is formed when the amine groups of chitosan and the carboxyl groups of other polymers interact electrostatically in a strong manner. As a consequence of being stronger than pure chitosan, the complex shows a higher activity under extreme mechanical stirring and temperature [39].

### 3.2. Alginate

Alginate is the most frequently used polymer for enzyme encapsulation [40]. It is a naturally derived polymer primarily found as a structural component of marine brown seaweed (*Laminaria hyperborea* and *Macrocystis pyrifera*) and as capsular polysaccharides in some soil bacteria such as *Pseudomonas* and *Azotobacter* [41]. In general, alginate is a linear polysaccharide copolymer composed of two C5 epimer repeating units, (1–4)-linked β-d-mannuronic acid (M units) and α-l-guluronic acid (G units) monomers. Within the alginate polymer, the M and G units are sequentially assembled in either repeating (-M-M- or -G-G-) or alternating (-M-G-) blocks [42]. The amount and distribution of each unit depend on the alginate source, and these blocks determine the polymer properties and behavior. The ratio between M and G units significantly affects transmittance, viscoelasticity, and swelling [43]. The carboxylic groups in alginate can form salts, such as sodium alginate, by their attachment to the monovalent ions.

Alginate can be prepared in either neutral or charged form, and so it is compatible with a broad variety of substances. Depending on media pH, alginate can form two types of gel, an acid or an ionotropic gel, which provide many physicochemical properties [44,45].

An important feature of alginate and its derivatives is its gelation in the presence of divalent cations such as calcium (Ca^2+^) through the ionic interaction between these cations and the carboxyl groups located on the polymer backbone. The most widely used cation for alginate hydrogels preparation is calcium, since it is an essential element for humans and is easily accessed [46]. The alginate gelation by calcium ions is due mainly to the ionic crosslinking between guluronic acid units located on adjacent alginate chains. This solution-gel transition process is called crosslinking. The crosslinked hydrogel has an “egg-box” structure.

Alginate hydrogels are nontoxic and immunologically inert materials with a high level of biocompatibility and biodegradability. They can easily undergo gelation with divalent cations under mild conditions suitable for the incorporation of biomacromolecules. Alginate-based microencapsulation is currently a favored approach for enzyme encapsulation [47,48]. A relatively simple and safe technique to entrap enzymes is the use of calcium alginate beads [49,50,51]. An aqueous solution of sodium alginate is mixed with the enzyme when added drop-wise in a solution of Ca^2+^, the droplets can precipitate, and the biocatalyst is entrapped [52]. In a 2% solution, calcium alginate beads are formed, resulting in 80 to 100 Å of average pore diameter [53,54,55]. The carboxyl groups of alginate provide multiple sites for ionic crosslinking with the positively charged amino acids present on enzymes structure. The enzyme and the support interact, improving the conformational stability of the immobilized enzyme and then increasing the shelf life [56]. According to Pereira et al. [57], sodium alginate titer can influence the enzyme entrapment immensely. At low concentrations (<1%), the viscosity of this polymer is low, which can cause internal mixing of the components during complexation, delaying the formation of a semipermeable surface that reduces enzyme retention. At higher concentrations (>1%) of this polymer, the enzyme retention increases, but above 4% (*w/v*), a small reduction in immobilization yield is observed, which is associated with viscosity increase. It is more difficult to extrude through the syringe needle when the viscosity is high, and, consequently, the formation of microcapsules is not uniform. Zusfahair et al. [58] studied the immobilization of amylase from *Aspergillus oryzae* and observed a reduction in immobilization yield while increasing alginate concentration. Conformational changes in the entrapped enzyme and limitation of substrate mass transfer to the microcapsules can be caused by increasing sodium alginate concentration.

Low mechanical strength, high leakage of the enzyme from beads, and large pore size are some disadvantages [53,54,59] which may be reduced by blending alginate with chitosan [57,60,61,62,63], kaolinite clay [64,65], gelatin [66,67], poly-l-lysine [68], and other polymers. The crosslinking of anionic alginate with cationic compounds results in a more controlled microcapsule pore size and stability improvement [67].

### 3.3. Carrageenan

Carrageenan is the name for a family of gel polysaccharides formed by a sulfated polygalactan that contains ester sulfate at about 15–40%. It is formed by alternating units of 3,6-anhydro-galactose and d-galactose, joined by β-1,4 and α-1,3 glycosidic linkage [69]. Carrageenan is extracted from red seaweed and can present various beneficial effects due to the variability of its structure and properties. The number of sulfate ester groups in a carrageenan molecule and its position, as well as the content of 3,6-anhydro-galactose, can influence the properties of the biopolymer [70].

Carrageenan has been produced in six types (κ-, ι-, λ-, μ-, ν-, and θ-carrageenan) based on their structures [71]. Due to its high gelling ability, κ-carrageenan is the most produced polymer. The enormous number of –OH supports the formation of the helix structure, forming many hydrogen bonds [72]. Gels produced with κ-carrageenan are hard, strong, and brittle. According to Geonzon et al. [73], the generally accepted model of the gelling process of carrageenan solutions involves the coil-to-helix transition, followed, in the presence of certain cations, by the aggregation of double helices to form a spanning network.

Zheng et al. [74] investigated the potential of carrageenan hydrogel beads for encapsulating β-galactosidase. The hydrogel beads were formed by injecting a β-galactosidase/κ -carrageenan solution into a potassium chloride hardening solution. The cationic potassium ions (K^+^) promote gelation of the anionic κ-carrageenan molecules by acting as salt bridges. In comparison with the free enzyme under certain thermal and pH conditions, the activity of encapsulated β-galactosidase was raised. In a recent study, pectin, carrageenan, and their hybrid hydrogels were investigated using the ionotropic gelation method for the encapsulation of β-galactosidase. As a result, the carrageenan hydrogel presented the best stability after three months, and its activity and release were considered better than that of the pectin hydrogel [75].

Much research on enzyme entrapment uses a κ-carrageenan as a wall material, such as for the entrapment of invertase [76], alpha-amylase [49], papain [50], urease [77], pancreatin [78], lactase [79], lipase [80,81], and glucoamylase [82].

### 3.4. Pectin

Pectin is a complex mixture of polysaccharides extracted from plant cell walls. Commercially available pectins are almost totally derived from citrus peels or apple pomace, which are by-products from the juice industry. Like the majority of other plant polysaccharides, it is polymolecular and polydisperse. In addition, its composition can vary depending on the source and the conditions faced during isolation [83].

Pectin consists mainly of D-galacturonic acid units, joined in chains through α-(1-4) glycosidic linkage. Pectin also has branch regions consisting of mono-sugars, such as d-xylose, d-glucose, l-rhamnose, l-arabinose, or d-galactose [84]. Some of the carboxyl groups of the uronic acids present in pectin chains are naturally present as methyl esters, and others are commercially treated with ammonia to produce carboxamide groups. The percentage of D-galacturonic acid esterified with methanol is denominated by the degree of methoxylation. According to the degree of the methoxylation, pectin can be classified into two groups: high methoxy pectin (HMD), with a degree of methylation > 50%, and low methoxy pectin (LMD), with a DM <50% [85].

Pectin has been employed as a gelling agent, a thickening agent, and a colloidal stabilizer for the last two centuries and is extensively applied in the food and pharmaceutical industries [86]. Due to its excellent gelling properties, pectin has been employed as a support material for enzyme entrapment. In an acidic environment (typically, pH ≤ 3) or with a high concentration co-solute (e.g., sucrose, ≥ 65 wt%), the gelation of high methoxy pectin occurs through crosslinking between the hydrophobic forces and methyl bonds [87].

Calcium dependent gelation is one of the most important functional properties of pectin that allows for enzyme entrapment. In this case, low methoxy pectin forms a gel in the presence of calcium ions. The gelation mechanisms of alginate and pectin, known as the egg-box model, were believed to be similar because their Ca-binding sites show a mirror-symmetric conformation. Nonetheless, the formation and the structure of egg-box dimmers between alginate and pectin differs, as studies have found [85]. Molecular modeling showed that the most well-disposed association of pectin chains should demonstrate a better correlation if described by a “shifted” egg-box [88].

In general, in order to keep a biocatalyst entrapped inside the pectin hydrogel network, pectins are used in the form of microbeads. The lipase encapsulation in pectin gels crosslinked with calcium ions increased enzyme activity by three to four times in water-miscible organic co-solvents compared with aqueous systems [89]. In another study, two enzymes were immobilized in pectin, presenting greater thermal and pH stability in comparison to the free enzyme system with the complete retention of original activities [90]. The bioactivity of the enzyme encapsulated depends on bead formulation and process parameters. β-lactamases encapsulated in pectin beads mainly depend on formulation parameters such as pectin type, CaCl_2_ concentration, and washing and drying processes [91].

Different hybrid supports based on pectin were described for enzyme encapsulation, such as a pectin/alginate [92,93,94], pectin/poly-vinyl alcohol [95], pectin/chitosan [96], carrageenan/pectin [75], and pectin/pine fiber [97]. The pectin combination with other polymers improves some key properties, such as mechanical and thermal resistances.

### 3.5. Agar–Agar and Agarose

Agar–agar is a natural polysaccharide obtained from the cell wall of *Rhodophyta* (red algae), and its main components are neutral agarose and charged agaropectin [98]. Agarose, the predominant component, is formed by agarobiose that consists of repeating units of β-D-galactose and 3,6-anhydro-l-galactose linked by the α-(1→3) and β-(1→4) glycosidic bond [99]. Agaropectin, a sulfated polysaccharide (3–10% sulfate), has the same repeating unit, but about 8% of the 2- or 6-positions of the 3,6-anhydro-α-l-galactose residues can be substituted by –OSO_3_, –OCH_3_, glucuronate, or pyruvate residues. The gelling potential of the material is greatly affected by these substituents [100].

Agar–agar and agarose gels have been used as a wall material for enzyme encapsulation because of some favorable functional properties [101,102,103,104,105]. Agarose, when in aqueous media, is a typical strongly hydrophilic, inert, and lyophilic colloid. Moreover, the ability of agar and agarose to form firm and stable gels is their most appealing characteristic [106].

Due to its physico-chemical properties and molecular structure, agarose transits into sol-gel after cooling and forms a three-dimensional network [107]. To form double helices, the single chains first associate via hydrogen bonds during gelation under cooling conditions. When the temperature drops even further, the double helices aggregate [108]. A two-step gelation mechanism was proposed: firstly, the connection between the randomly distributed coils by hydrogen bonds is formed, and a double-helical association is made; then, the double helices aggregate, forming a tight, three-dimensional network [109]. Moreover, the coil-to-helix transition, which occurs while cooling, may be described by a mean-field Zimm–Bragg approach [110].

The pore size of the gel matrix is regulated by the agarose concentration, which affects the immobilization yield of the enzyme and its catalytic performance. A fragile gel matrix with large pores is produced with a lower concentration, leading to the leaking of enzyme molecules. In contrast, small pore size within the gel matrix is caused by high concentration, creating hurdles in the penetration of high molecular weight substrates [111,112]. The maximum immobilization yield (%) of the enzyme was achieved when 2.0% agarose was used, according to Karim et al. [113]. Through an entrapment technique, these authors encapsulated carboxymethyl cellulase from *Bacillus licheniformis* KIBGE-IB2 within the agarose gel matrix. After it was immobilized, the enzyme’s activation energy (Ea) increased from 16.38 to 44.08 kJ/mol. The immobilized enzyme exhibited higher catalytic activity in a broad range of pH and temperature as compared to native enzymes. Furthermore, the operational and storage stabilities were also found to be significantly higher when the enzyme was immobilized.

The agar–agarose blend was used as a wall material for α-amylase entrapment [114]. Maximum immobilization yield (19.9%) was obtained with beads prepared with 1.0% (*w/v*) agarose and 4.0% (*w/v*) agar. The immobilized enzyme had a hydrolytic activity nearly 25% higher when compared to that of the free enzyme.

Serine protease produced by *Aspergillus niger* KIBGE-IB36 was encapsulated in agar–agar hydrogel, and maximum enzymatic activity was attained when 3.0% agar–agar was used. The immobilized protease exhibited a significant increase in the thermal stability and retained approximately 68.0% of its residual activity at 60 °C. As the entrapped protease showed enzymatic activity, storage stability increased up to 30 days compared to the soluble enzyme. The enzyme was recycled up to eight cycles, presenting an exceptional attribute for economic utility and the continuous recycling of protease [115].

### 3.6. Gelatin

Gelatin is a protein of animal origin that is derived from the chemical degradation of collagen. Cattle bones, hides, pig skins, and fish are the main commercial sources of gelatin and can be obtained inexpensively. It has a high molecular weight from 65,000 to 300,000 g/mol [116]. Chemically, gelatin comprises 18 varieties of complex amino acids, with glycine, proline, and hydroxyproline as the major compounds, and other distinguished amino acid families, such as glutamic acid, alanine arginine, and aspartic acid [117]. Due to its excellent biodegradability, low cytotoxicity, and indefinite shelf life, gelatin has attracted attention for enzyme immobilization [118].

Gelatin can form a heat-reversible gel in dilute aqueous solutions [119] and a hydrophilic, macroporous hydrogel containing hydroxyl groups as well as charged groups (–NH_2_, –COOH). All these available groups on the molecular chains can be activated and then covalently conjugated with polymeric gelatin chains by crosslinking agents such as microbial transglutaminase [120]. Transglutaminase presents a singular ability of protein crosslinking, catalyzing acyl-transfer reactions between γ-carboxyamide groups of glutamine residues and ε-amino groups of lysine residues [121]. According to Labus et al. [67,122], gelatin-based hydrogels crosslinked with transglutaminase are suitable to be used as matrices for invertase and β-galactosidase entrapment.

Physical, enzymatic, and chemical methods can be used to develop an efficient procedure to prepare gelatin hydrogels of appropriate strength and elastic properties for applications as enzyme carriers. Generally, gelatin microspheres are prepared primarily using (1) spray-drying, (2) coacervation, (3) emulsion, or (4) membrane emulsification methods [118,123,124]. The immobilization of enzymes onto gelatin takes place via crosslinking between the enzyme molecule with crosslinking agents and the free amino groups of the carrier, leading to the formation of a covalent bond, according to Ewadh and Al-Khafaji [125]. Gan et al. [126] elaborated a glucoamylase-immobilized system based on crosslinked gelatin nanoparticles using the coacervation method. This system exhibited characteristics of temperature-triggered phase transition, which could be used for enzyme immobilization and release. The efficiency of the loadings of immobilized glucoamylase by entrapment was 59.9%. The immobilized enzyme was released when the system temperature was above 40 °C.

Gelatin behaves like an amphoteric electrolyte in solution (carrying a net positive charge below its isoelectric pH). It is also known to interact via non-covalent interactions with other hydrocolloids, including alginate, gellan, carrageenan, and konjac glucomannan [127]. Efficient enzyme immobilization includes laccase [128], β-glucosidase [129], α-amylase [130], and lipase [131] by using gelatin along with calcium alginate, polyester films, and titanium species [132].

### 3.7. Polyethylene Glycol (PEG)

Polyethylene glycol (PEG) is a hydrophilic polymer composed of repeated ethylene glycol units [–(CH_2_CH_2_O)_n_]. It can be synthesized by anionic polymerization of ethylene oxide and a hydroxyl group (from water, ethylene glycol, or any diols). Ring-opening polymerization of epoxyethane is another way to produce PEG. Commercially available PEGs are found with different degrees of polymerization and activated functional groups [133].

From the synthetic polymers, polyethylene glycol stands out as an encapsulating material for enzyme systems because of its non-immunogenic, biocompatible, and flexible nature [134]. The mechanical stability of PEG can be enhanced by mixing it with other polymers, such as alginate, chitosan [135], and poly (lactic-*co*-glycolic acid) (PLGA) [136].

Wang et al. [135] showed that PEG added into sodium alginate-immobilized cellulase increased the matrix porosity. The addition of chitosan reduced the disintegration of the carrier to improve its stability. Cellulase immobilized in a sodium alginate–PEG–chitosan matrix was used to hydrolyze microcrystalline cellulose with an overall yield 23% higher than that of the free cellulase after reusing it for five cycles.

Labile hydrophilic enzymes for neurological disease applications can also be delivered by polymeric particles made of PEG. PLGA copolymerized with PEG has been used to protect catalase from degradative proteases [136]. Sonicated nanoparticles of PEG–PLGA containing catalase increased the enzyme activity and showed great protection of the enzyme in degradative conditions. The authors, however, were alert for possible toxicity caused by the solvent used during sonication. Replacing dichloromethane with chloroform resulted in biocompatible polymeric nanoparticle formulations [136].

PEG was also used to encapsulate laccase for bisphenol A removal from aqueous solution [137]. The particles were prepared by encapsulating laccases into PEG hydrogel via the UV-assisted emulsion polymerization method followed by cross linking with glutaraldehyde. High enzyme entrapment efficiency and activity recovery were obtained, resulting in successful bisphenol degradation for seven cycles.

## 4. Enzyme Encapsulation Methods

The selection of the encapsulating method is of great importance for the success of micro or nanostructured systems for enzyme loading. In recent decades, several methods have been studied for this purpose, especially ionic gelation, spray drying [16,57], freeze-drying [138,139], nanoprecipitation [18,140], and electrospinning [19,141]. A good encapsulation method should enable high loading capacity, high encapsulation efficiency, and high stability. The release and bioavailability characteristics of the capsule must also be considered for enzyme delivery [142,143].

Table 1 shows some recent studies of enzymatic encapsulation using polymers, and the main results are reported.

As can be seen in Table 1, numerous techniques can be used in enzyme encapsulation, especially ionic gelation, spray drying, freeze-drying, nanoprecipitation, and electrospinning. α-chymotrypsin, lysozyme, and β-galactosidase are the most used enzymes in encapsulation studies with polymeric matrices (Table 1). The most prominent polymers are chitosan and sodium alginate, which may be related to the abundance of these polymers in nature. Furthermore, most authors reported high encapsulation efficiency [16,19,57,138,140,152,154], improvement or maintenance of enzyme activity [16,19,57,138,144,148,151,152,162], improved pH, temperature or storage stability [150,151,152,158,161], and high reuse capacity [132,163]. The most widely used methods for entrapping enzymes are described in the next sections.

### 4.1. Ionic Gelation

**Figure 1:**
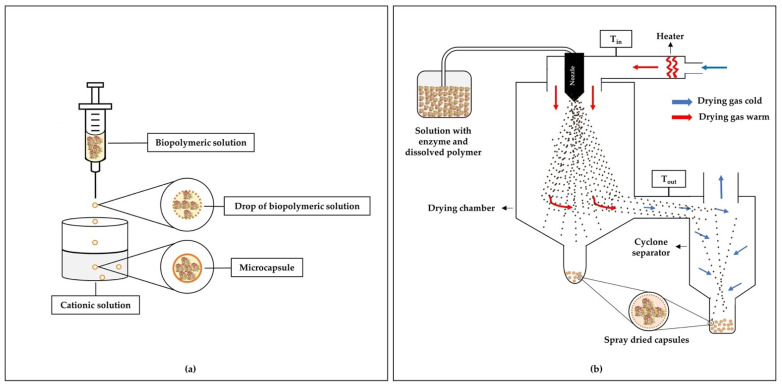
Schemes for encapsulation of enzymes using polymers: (**a**) ionic gelation method and (**b**) spray drying method.

Ionic gelation is often used to prepare micro/nanoformulations for the controlled release of enzymes. In this method, solutions of biopolymers (e.g., alginates, carboxymethylcellulose, chitosan) are dripped/sprayed under constant agitation to solutions containing divalent or trivalent cations (for example, Ca^2+^, Sr^2+^, Ba^2+^, Al^3+^), which induce gelation [14]. Encapsulation occurs by dissolving the enzyme in the polymeric solution before the microparticle formation process. Ionic gelation is a simple and easy method, does not require specialized equipment, organic solvent, or high temperatures, and can be considered low cost [164,165]. Figure 1a illustrates an example of enzyme encapsulation by the ionic gelation method.

Studies carried out by Bahreini et al. described the immobilization of L-asparaginase on chitosan nanoparticles by the ionotropic gelling method using tripolyphosphate as anion [166]. The authors reported that the best chitosan/tripolyphosphate ratio was 4.2, with an encapsulation efficiency of 76.2%. In addition, the immobilized enzyme showed an increase in half-life of about 23 days in the low ionic strength solution compared to the free enzyme. Vimal and Kumar [167] also immobilized L-asparaginase in chitosan nanoparticles by the ionic gelling method and obtained an encapsulation efficiency and loading capacity of 72% and 53%, respectively. Encapsulation of l-asparaginase inside the nanocarrier improved its pH and thermal stability.

Degradation studies of various textile dyes with horseradish peroxidase encapsulated in chitosan granules were evaluated by Bilal et al. [168]. In this study, the horseradish enzyme was effectively immobilized in chitosan granules (2.5% chitosan concentration) with an immobilization yield of 92.54% using a simple entrapment method. The optimum pH and temperature of the immobilized enzyme were 7.5 and 70 °C, respectively. The dye discoloration potential by immobilized peroxidase was investigated in a fixed bed reactor system for four different textile dyes, namely Remazol Brilliant Blue R (RBBR), Reactive Black 5 (RB5), Congo Red (CR), and Crystal Violet (CV). Immobilized peroxidase resulted in effective dye removal of RB5 (97.82%) followed by CR (94.35%), CV (87.43%), and RBBR (82.17%). The immobilized peroxidase retained up to 64.9% of the residual activity after six consecutive cycles of dye decolorization.

Jaiswal et al. [169] immobilized a purified papaya laccase on chitosan granules using an entrapment approach. Papaya laccase was immobilized in chitosan granules with an immobilization yield and loading efficiency of 98% and 100%, respectively. An increase in laccase properties, such as optimal temperature (at 10 °C), thermostability (by 3 times), and optimal pH (from 8.0 to 10.0), was observed after immobilization. Immobilization increased enzyme tolerance to a range of metal ions (including heavy metals) and organic solvents, namely, ethanol, isopropanol, methanol, benzene, and dimethylformamide (DMF).

### 4.2. Spray Drying

Spray drying is widely used to encapsulate active ingredients, such as flavonoids, lipids, carotenoids, and enzymes [16,157,170]. Spray drying is a continuous process that transforms various liquids (for example, solutions, emulsions, dispersions, slurries, pastes, or even melts) into solid particles with adjustable size, distribution, shape, porosity, density, and chemical composition [171]. In addition, it is a simple, fast, and relatively low-cost process.

In the encapsulation using the spray drying technique, the feed material is atomized inside the drying chamber, in which the water of the formed droplets is instantly evaporated due to contact with the hot air inside the chamber. The formed microparticles are then separated from the drying air using recovery cyclones [172]. The short contact time (a few seconds) of the heat with the formed particles makes spray drying encapsulation quite attractive for enzyme trapping. In addition, proteins and enzymes are more resistant to degradation by heat in conditions of low water content and are stabilized very quickly during rapid evaporation [173]. Figure 1b shows a schematic of a traditional spray drying system used in the production of microparticles.

Estevinho et al. [157] encapsulated β-galactosidase using the spray drying method and chitosan as an encapsulating agent and observed an increase in the diffusional effect of the released enzyme and also a reduction in the initial enzyme activity. Ataide et al. [138] performed the encapsulation of bromelain in chitosan nanoparticles, aiming to reduce degradation by protease, increasing its stability and efficiency. The chitosan–bromelain nanoparticles were produced by ionic gelation/freeze-drying, and an encapsulation efficiency of 85% and an enzymatic activity of 4.9 U/mL were observed.

### 4.3. Freeze-Drying

Freeze-drying is an appropriate and widely used technique for microencapsulation of heat-sensitive compounds, as it removes water from microparticles by sublimation. It is a multi-stage operation that is divided into three stages: freezing, sublimation, and desorption. The freezing step converts most of the sample water to ice and then the ice is removed by sublimation at a very low temperature and pressure (step 2). Finally, the non-frozen water is desorbed to the desired moisture content [174].

**Figure 2:**
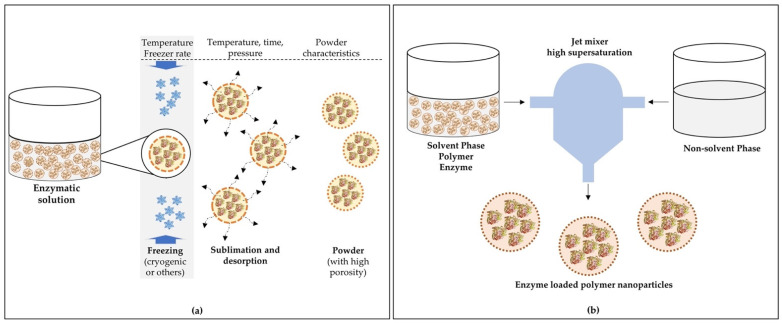
Schemes of capsule production loaded with enzymes by (**a**) a freeze-drying process and (**b**) the flash nanoprecipitation method. Adapted from Vishali et al. [175] and Martínez Rivas et al. [176].

The freeze-drying process alone is not capable of producing microparticles and, therefore, before this process, the enzyme needs to be encapsulated by another method that can form droplets of the desired size (e.g., emulsification), which are subsequently dehydrated by lyophilization, forming micro- or nanocapsules. Figure 2a shows an example of powdered nanocapsules containing enzymes produced using freeze-drying.

Freeze-drying can also be applied as a complementary technique. Using the ionic crosslinking technique, the encapsulation of bromelain in chitosan nanoparticles resulted in high encapsulation yield and high enzymatic activity [138]. However, the particles were unstable in aqueous media, and lyophilization of the formulation with glycine and maltose as lyoprotectants was evaluated. The resulting microparticles showed short resuspension time, little changed average size, and increased encapsulation rate compared to the liquid form. Maltose has been identified as the best lyoprotectant for the maintenance of enzyme activity, especially when stored under refrigeration [138].

Firefly luciferase was encapsulated in a freeze-dried hydrogel prepared from a colloidal suspension of chitosan and xanthan gum. The enzyme was successfully stabilized and released from the rehydrated samples at a moderate rate. Furthermore, the enzymatic activities of the encapsulated and released enzyme were confirmed for more than 30 days, which reinforces the potential of this technique for enzyme protection [139].

### 4.4. Nanoprecipitation

Nanoprecipitation is a simple and easy technique to reproduce, and it is commonly used to prepare nanoparticles [176]. This method is based on the interfacial deposition of a polymer following the displacement of a semi-polar solvent miscible with water from a lipophilic solution [177]. The process consists, initially, of the preparation of the solvent phase, normally composed of the film-forming polymer, one or more molecules to be encapsulated, a lipophilic surfactant, and one or more organic solvents, and the non-solvent phase, usually consisting of water. Subsequently, the two phases are mixed under moderate magnetic stirring, and the organic solvent is evaporated at room temperature or with a rotary evaporator, resulting in the nanoparticles being in an aqueous suspension. The water in the aqueous suspension containing the nanoparticles can be removed by ultracentrifugation or freeze-drying [176]. Figure 2b shows a scheme to produce nanocapsules containing enzymes by the nanoprecipitation method.

Several polymers can be used in the encapsulation process by nanoprecipitation, emphasizing biodegradable polyesters such as polylactide, polylactide-*co*-glycolide, and poly-e-caprolactone. Likewise, several solvents have been used in this process, including ethanol, acetone, hexane, methylene chloride, or dioxane [176].

Markwalter et al. developed polymeric nanocarriers composed of poly (lactic acid) and poly (ethylene glycol) using the technique of inverse flash nanoprecipitation and demonstrated the wide applicability of the process, a large load capacity, and high reproducibility when encapsulating lysozyme and horseradish peroxidase [18]. Encapsulation retained 99% of enzyme activity after processing. Asparaginase was also encapsulated by nanoprecipitation in polyglycerol nanogels with an efficiency of almost 100% [140]. Furthermore, no structural changes were observed after exposure to an acid environment to promote enzyme release, and the total enzymatic activity was maintained.

### 4.5. Electrospinning

**Figure 3:**
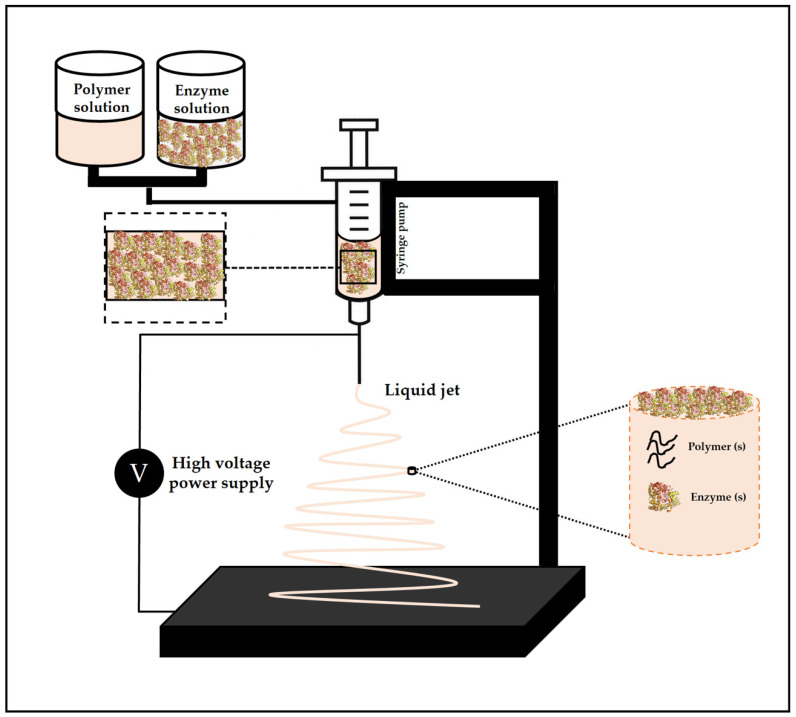
Basic diagram of the electrospinning process.

Electrospinning is used to prepare continuous fibers on a sub-micron or nanometric scale through the action of an external electric field [178,179]. This process is carried out using a polymer or melted polymer solution, which is usually pumped using a syringe needle, to which a high voltage is applied [180]. The applied voltage induces a charge movement in the polymeric liquid, capable of stretching the droplet’s shape, which normally is a sphere formed by the surface tension [180]. Once the electrostatic repulsion of the charged liquid polymer becomes greater than the surface tension, a conical shape known as Taylor’s cone is formed, and the jet initiation begins at the tip of the cone [180]. If there is sufficient cohesive force in the polymer liquid, a stable stream is ejected from the Taylor cone, allowing the polymer chains to stretch together and form a uniform filament, which is deposited on a grounded metallic collector [180]. A typical electrospinning system is shown schematically in Figure 3.

Gabrielczyk et al. [141] evaluated the encapsulation performance of *Bacillus subtilis* fructosyltransferase by coaxial electrospinning using a set of biodegradable polymers and obtained an enzyme load of 68.1 mg/g and a specific enzyme activity of 5.5 U/mg. An electrospun double-layer mucoadhesive patch was proposed by Edmans et al. [19] to deliver proteins to the oral mucosa. Lysozyme was incorporated into poly (vinylpyrrolidone)/Eudragit RS100 polymer nanofibers using electrospinning. A high encapsulation efficiency and preservation of enzyme activity were achieved (93.4% and 96.1%, respectively).

## 5. Release Mechanisms for Encapsulated Enzymes

**Figure 4:**
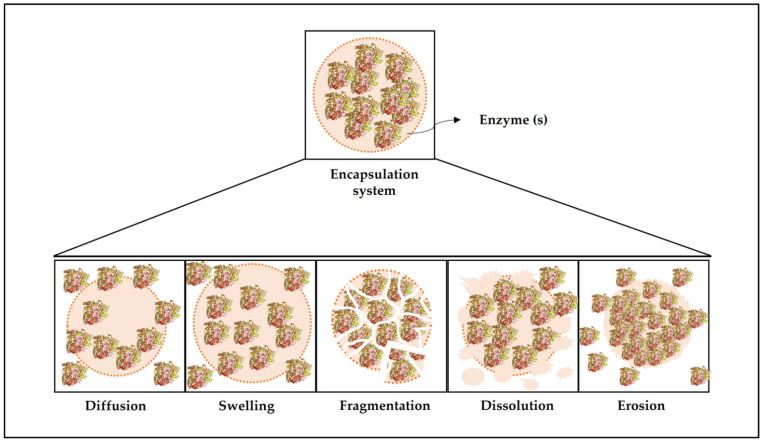
Different mechanisms of enzyme release from polymeric matrices.

Delivery systems are designed to release the active ingredients at a given site of action, at a controlled rate, or in response to a specific environmental trigger such as pH, ionic strength, temperature, or enzyme activity [8]. In most applications of encapsulated enzymes, controlled release is essential to obtain the desired effect, as it guarantees a hydrolysis- or synthesis-controlled profile. Several release mechanisms have already been studied, and the most common are discussed below and represented in Figure 4.

Diffusion is the dominant mechanism in delivery systems [8], which consists of the random movement of the active molecules due to the existence of a chemical concentration gradient [181]. The rate at which the active ingredient is released during the diffusive process depends on many factors, including solute properties (such as molecular weight and polarity), matrix properties (such as polarity, rheology, physical state, and interactions), characteristics of the microparticle (such as size, shape, and structure), and the gradient of solute concentration between the particle and the surrounding environment. Moreover, the encapsulating system may be stable throughout diffusion or undergo changes due to events of swelling, shrinkage, erosion, and fragmentation [8].

Release by the swelling mechanism is also quite common. In this mechanism, the active ingredient is released from the particles when they absorb the solvent from the surrounding medium (usually water) and swell, increasing the pores’ size. The release rate of the active ingredient will depend on the swelling rate and the time it takes the active ingredients to diffuse through the swollen microparticle matrix. [8]. The swelling release can be controlled by selecting the appropriate polymeric matrix, as well as the conditions of the surrounding environment, including temperature and pH [9]. The release can also occur by fragmentation, when the microstructure breaks due to external conditions, such as mechanical pressure, shear, pH changes, and others [9]. The release rate in this mechanism depends on the fracture properties of the particle and the size and shape of the fragments formed. Normally, the release by this mechanism is rapid due to the increase in surface area and the influence of other release mechanisms, such as diffusion, dissolution, and erosion [8].

There are also encapsulation systems that use the dissolution release mechanism, considered one of the simplest designs [182]. Release by this mechanism occurs when the encapsulating system is placed under conditions that lead to the dissolution of the microstructure. The microstructure can be completely constituted by the active molecule, releasing the product as the particle dissolves, or it may consist of a polymeric matrix containing the dispersed active material, in which the release occurs by the dissolution of that matrix [8]. The release rate by this mechanism is directly correlated to the dissolution rate, which depends on the microparticle’s composition and structure and external factors, such as pH, solvent type, ionic strength, and temperature [8]. Finally, delivery can also occur through the erosion mechanism, which consists of the erosion of the microparticle matrix due to physical factors, such as temperature; chemicals, such as strong acids or bases; and enzymes, such as lipases and amylases. The erosion process can occur superficially or fully, and the release profile is directly related to the erosion rate. The greater the erosion, the greater the release rates of the encapsulated molecules [8,9].

Babich et al. [156] evaluated the release of L-phenylalanine ammonia-lyase encapsulated in simulated fluids and observed that, in distilled water, the degree of degradation of the capsules containing the enzyme reached 55–65% after 120 min. The maximum degradations of the microcapsules (90–98%) occurred in a bio-relevant media model simulating intestinal juice. Osman et al. [16] evaluated the in vitro release of DNase I from a microparticle system to treat cystic fibrosis via the pulmonary route and observed that for microparticles without surface modification, about 24% of the enzyme was released during the first 6 h. The inclusion of the hydrophilic surface modifiers increased this amount to varying degrees, which corresponds to the DNase I located on the surface of the particles.

The release rate of firefly luciferase encapsulated in the freeze-dried hydrogel of chitosan and xanthan gum was investigated using a standard leaching (erosion) test. The enzyme release rate was higher at pH 6.0, and the addition of montmorillonite nanoclay significantly reduced the rate of enzyme release due to the strong influence on the structural modification of the bionanocomposites [139]. Anjani et al. [14] evaluated the in vitro release rate of flavourzyme by the dissolution mechanism using a trisodium citrate buffer at different concentrations (0.5–2.0%). The authors found that the release rate increased with an increment in buffer concentration from 0.5 to 2.0%.

## 6. Characterization of Encapsulation Systems

The characterization of microparticles is crucial in the overall encapsulation process because it provides important information for process optimization. The main techniques used to characterize encapsulation systems developed for the trapping of enzymes are usually aimed at evaluating the yield and efficiency of encapsulation, morphological characteristics, and physical aspects [13].

Yield and efficiency encapsulation are variables that are related to the quantification of the active molecule incorporated in the microstructures. These parameters can be established by analytical methods, such as UV-visible spectrophotometry, high-performance liquid chromatography (HPLC), or gas chromatography (GC) [177]. The determination of yield and encapsulation efficiency is essential in enzyme trapping because the encapsulation yield may not positively correlate with the encapsulation efficiency, that is, obtaining a high encapsulation yield may not provide a high encapsulation efficiency. This is because the encapsulation efficiency is linked to the concentration of active molecules after the encapsulation process, which does not occur for the encapsulation yield, which is associated with the total concentration of the molecule (active or inactive). These facts can be observed in the study developed by Pereira et al. [57]. They found a high encapsulation yield for *Yarrowia lipolytica* lipase (>90%) in an alginate and chitosan matrix but noted that the enzyme activity was low in some cases (low encapsulation efficiency).

The characterization of the particles produced in terms of surface size and morphology is also quite relevant. Scanning electron microscopy (SEM), transmission electron microscopy (TEM), and dynamic light scattering (DLS) are the techniques most used for this purpose [183,184]. Pereira et al. [13] used SEM to evaluate the morphology of microcapsules containing *Yarrowia lipolytica* lipase obtained by the ion gelation technique and observed a collapsed and heterogeneous surface after drying by freeze-drying. This observation was necessary for the authors to understand the significant increase in enzyme activity after the drying process was attributed to the increase in the surface area.

The evaluation of the physical characteristics of the microparticles are also crucial, and the main ones are zeta potential, thermal gravimetric analysis (TGA), differential thermal analysis (DTA), differential scanning calorimetry (DSC), Fourier transform infrared spectroscopy (FTIR), and X-ray diffraction. Zeta potential determines the surface electrical charge of the microparticles and is of great importance in assessing their stability and behavior in a biological environment [13,177]. TGA, DTA, and DSC are thermodynamic characterizations that provide important information about the properties of the chemical compounds that form the microparticles. TGA uses heat to force reactions and physical changes in materials and generate thermogravimetric curves that characterize specific compounds due to the unique sequence of physical–chemical reactions. DTA is based on the principle that the substance, when heated, undergoes reactions and phase changes that involve absorption or heat emission, with the identification of a substance being performed by comparing the DTA curves obtained from the unknown substance with the DTA curves that are provided by elements [177].

On the other hand, DSC is based on the release of heat from a chemical process, which is determined by the variation of enthalpy [185]. Another important analysis in the physical characterization of the microparticles is the FTIR. This analysis is used to evaluate the possible chemical interactions between polymers and biomolecules. Finally, another analysis of equal importance is X-ray diffraction, normally used to verify the crystalline and amorphous regions of the microparticles [13].

## 7. Factors Influencing Active Molecules Encapsulation Efficiency

Different parameters can affect the rate of solidification of microparticles, directly influencing the encapsulation efficiency. The encapsulating system must ensure the delivery of enzymes in their native folding state and structure so that their biological activity is sustained [186]. Nevertheless, the encapsulating process’ success depends on the characteristics of coating materials and the stability of active compounds in the core, along with the adequacy of the delivery system for its application [187].

Encapsulation efficiency can increase as the polymer concentration rises once the high concentration induces fast precipitation of the polymer on the surface of the dispersed phase, retarding the active molecule diffusion. Moreover, when diffusional resistance to encapsulated material from the organic phase to the aqueous phase is high, more active molecules are entrapped, raising particle size [188]. The viscosity of the solution increases when it is highly concentrated, also leading to encapsulating material dispersion delay. In addition, high viscosity and rapid solidification of the dispersed phase can diminish the microparticles’ porosity and consequently improve the encapsulating efficiency [189].

Srikar and Rani [17] indicated that the molecular weight of the polymer was directly proportional to the entrapment efficiency. For polymers that display high viscosity, the active molecule are encapsulated longer than in the presence of low molecular weight polymers. Encapsulation efficiency can fall within a certain particle size range, and the active molecule release rate can accelerate if the particle size is reduced. It was also observed that particle size can decrease when the organic phase’s concentration increases once the viscosity of the solution rises [189].

Polymer hydrophobicity can also influence the entrapment efficiency since the encapsulating material is highly hydrophobic; it hampers the encapsulated active molecule’s escape to the aqueous phase, achieving a low efficiency [190]. The opposite is observed when the entrapped molecule presents higher solubility in the continuous phase than in the dispersed phase, leading to an easy diffusion and good encapsulation efficiency [189].

Another factor influencing the encapsulation efficiency is the ratio of dispersed phase to continuous phase (D/C ratio). When the D/C ratio decreases, it was observed that active molecule loading and encapsulation efficiency increased and the microsphere surface was smoother, perhaps because of a faster solidification rate. The porosity in a system of microspheres can be established during encapsulating polymer hardening when the organic solvent evaporates during preparation. If a continuous phase contains a large amount of water, the polymer will precipitate faster, and consequently, less porous spheres will be formed. Additionally, it was reported that as the volume of the continuous phase rises, the size of microspheres reduces, promoting a decrease in loading efficiency and a faster molecule release [18].

Considering the interaction between polymer and active molecules, encapsulation efficiency can increase when molecules as proteins interact with polymers carrying free carboxylic end groups compared to end-capped ones. This occurs if electrostatic interactions are involved in the encapsulation process. However, protein release from the microparticles can be limited if the interaction is too strong. As an alternative, a co-encapsulated excipient can intermediate the interaction between the protein and polymer [186,189].

## 8. Applications of Polymer Encapsulated Enzymes

Encapsulated enzymes can be used for several different purposes, and therefore can find applications in a wide range of industrial sectors. Some examples that show pharmaceutical, medical, and food applications are depicted in Table 2. Despite the recent articles showing the use of encapsulating enzymes in biocatalysis [13,141], this use is not so intense because of the low reuse efficiency and reduced mass transfer. Pereira et al. [13] reported that p-nitrophenyl laurate hydrolysis by lipase encapsulated in chitosan–alginate beads decreased by 52% in the second reaction cycle and indicated a reduction of reaction rate, which might be due to substrate or product retained in the active site.

Drug delivery strategies that can be achieved with encapsulating enzymes are significantly advantageous for pharmaceutical and medical applications because they can direct the enzyme to the right location. Edmans et al. [19] incorporated lysozyme into poly (vinylpyrrolidone)/Eudragit RS100 polymer nanofibers using electrospinning from an ethanol/water mixture as an antimicrobial protein to the oral mucosa. The resulting fibrous membranes released the protein at a clinically desirable rate (90% cumulative release after 2 h) and inhibited the growth of the oral bacterium *Streptococcus ratti*, showing its potential to treat and prevent oral infections. Vimal and Kumar [167] reported that the antimicrobial property of l-asparaginase increased when it was encapsulated inside a chitosan nanoparticle, demonstrating that the enzyme nano-carrier has better therapeutic potential as compared to the free enzyme. Osman et al. [16] tested various hydrophilic excipients to produce controlled release microparticles by co-spray drying DNase I with poly (lactic-*co*-glycolic) acid and 1,2-dipalmitoyl-Sn-phosphatidyl choline (biocompatible surfactant). These microparticles prepared with dextran were biocompatible with lung epithelial cells and showed a controlled release to reduce cystic fibrosis mucus viscosity.

The incorporation of enzymes into food products can also be achieved with encapsulation strategies. Anjani et al. [14] used flavourzyme encapsulated with alginate for cheese ripening. The enzyme leakage from capsules increased with an increase in the duration of the simulated cheese press from 4 to 16 h, but the control of the amount of enzyme leakage is still needed to direct the proteolysis required for optimal flavor development in accelerated cheese ripening. Microbial pectinase encapsulated by sodium alginate was used successfully by Mahmoud et al. [163] to eliminate pectin from orange juice, reducing turbidity since the free enzyme cannot be used in this case due to the high acidity of the juice.

Another possible application of encapsulated enzymes is for polymerase chain reaction (PCR), which is an extensively used technique to amplify DNA in vitro. Due to the limitation of parallel reactions by the conventional methodology, major screening studies are restricted. Mak et al. [192] proposed the use of diffusion controlled and temperature stable microcapsule compartments to perform a high number of individual PCRs. The authors demonstrated that a great number of individual PCRs confined in microcapsules containing Taq DNA polymerase can occur in a single reaction tube since low molecular reactants are allowed to diffuse in the permeability controlled capsules.

**Figure 5:**
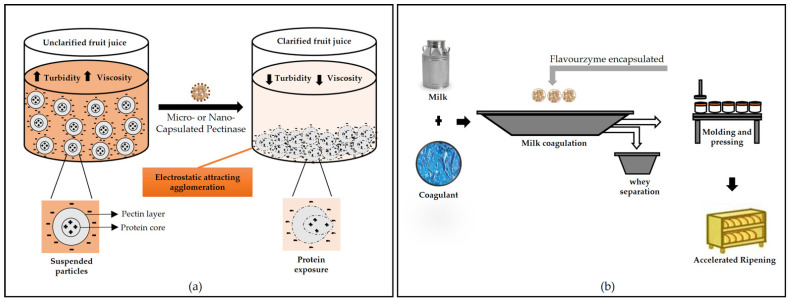

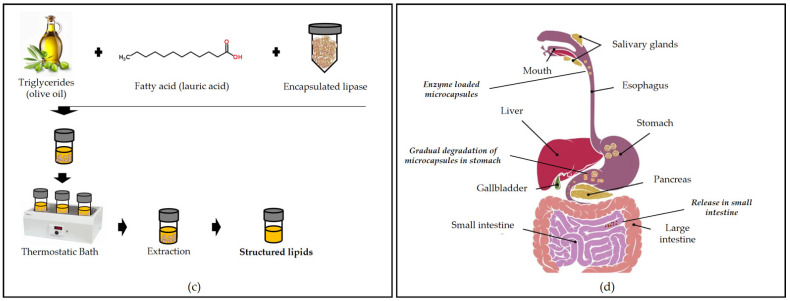
Application of microcapsules containing enzymes: (**a**) juice clarification, (**b**) cheese maturation, (**c**) biocatalysis of structured lipids, and (**d**) oral drug delivery.

RNA is a biomolecule with a wide range of important cellular functions, such as information storage, catalysis, and regulation, and can also benefit from encapsulation considering its susceptibility to degradation. RNA was successfully synthesized and internalized in polymer hydrogel capsules containing T7 RNA polymerase, leading to minimization of molecule handling and bypassing isolation and purification paths [193,194]. Figure 5 illustrates some possible applications of encapsulated enzymes.

## 9. Conclusions and Future Perspectives

When using enzymes, we are faced with one huge problem: their stability. The stability of enzymes is linked to interactions formed in their structure, which can be easily destabilized when placed in adverse environmental conditions, such as variations in temperature, pH, or ionic strength. Therefore, delivery systems, such as encapsulation with polymers, are an alternative way to protect the catalytic activity of enzymes until they reach the target site, where they will be released, or during their use in a reaction system. Ionic gelation, spray drying, freeze-drying, nanoprecipitation, and electrospinning are the main techniques reported for the development of enzyme delivery systems. The use of these transporters has shown great benefits in improving enzymatic stability, which increases the possibility of using these biomolecules in industry. However, only a few polymers have been tested so far, which shows that new options can be studied in future research. Other methods such as coacervation, double emulsion, and polymerization have been tested successfully in a few studies and should also be the targets of new insights. The development of new polymeric structures for the encapsulation of enzymes, aiming at their application and reuse in catalytic systems, should also be explored. The information covered in this review provides guidance for the future development of more advanced transport systems for application in enzyme encapsulation.

## Figures and Tables

**Table 1:** Recent research about the encapsulation of enzymes using polymeric matrices.

Encapsulation Method	Enzyme	Encapsulating Polymers	Main Results	Refs.
Liposome entrapment	Superoxide dismutase	Polyacrylamide	Good encapsulation efficiency (37%) and maintenance of enzyme activity.	[144]
Emulsion solvent evaporation	α-chymotrypsin and lysozyme	Poly (glycerol adipate-*co*-o-pentadecalactone) Poly (1,3-propanediol adipate-*co*-o-pentadecalactone)	Little difference in encapsulation was observed between the different polymers; Changes in polymer chemistry showed greater effects.	[145]
Crosslinked enzyme aggregates	Thioesterase, galactosylceramidase, α-glucosidase, and β- glucosidase	Poly (lactide-*co*-glycolide)	Excellent activity retention (usually around 60%); enzymatic activity is fully recovered in primary fibroblasts upon treatment.	[146]
Solid-in-oil-in-water	α-chymotrypsin	Poly (lactic-*co*-glycolic) acid	Maximum encapsulation efficiency of 61%.	[147]
Precipitation-dialysis	α-chymotrypsin	Poly (γ-glutamic acid)	Considerable amounts of α-chymotrypsin were encapsulated (20–25%); the encapsulation contributed to the preservation of enzyme activity over time.	[148]
Adsorption	Alcohol dehydrogenase	Polyallylamine Polystyrene sulfonate	The affinity of alcohol dehydrogenase to the substrate was 1.7 times lower than that of the native enzyme.	[149]
Polymerization	Glucose oxidase	-	Thermal stability and tolerance to organic solvents were significantly improved.	[150]
Homogenization	Naringinase	Sodium alginate or chitosan	The process improved the kinetics and operational stability, so it could be useful as a debittering agent for citrus juice industries.	[151]
Electrospinning	Lysozyme	Poly(vinylpyrrolidone) and Eudragit RS100	High encapsulation efficiency and preservation of enzyme activity were achieved (93.4 ± 7.0% and 96.1 ± 3.3%, respectively).	[19]
	Fructosyltransferase	Group of biodegradable polymers	Good results have been obtained; however, further research is needed to reduce the leaching of the encapsulated enzyme from electrophilized fibers.	[141]
	β-galactosidase	Polyvinylpyrrolidone	97% of the original activity was maintained; there were no changes in pH and temperature profiles; high storage stability (β-galactosidase activity decreased by only 4% after one year).	[152]
Electrospray	Streptokinase	Poly (lactic-*co*-glycolic acid)	The method proved to be an interesting approach to encapsulate enzymes; other studies are necessary to ensure the maintenance of enzyme activity after electrospray.	[153]
Freeze-drying	Bromelain	Chitosan	The freeze-dried method can effectively improve the stability of bromelain and nanoparticles.	[138]
	Firefly luciferase	Chitosan and xanthan gum	Enzymatic activities of the encapsulated and the released enzyme were confirmed for over 30 days.	[139]
Nanoprecipitation	Lysozyme and horseradish peroxidase	Poly (lactic acid) Poly (ethylene glycol)	Lysozyme and horseradish peroxidase were shown to retain 99% activity after processing.	[18]
	Asparaginase	Polyglycerol	Enzymes were encapsulated with an efficacy of 100% and, after release, full enzyme activity and structural integrity were retained.	[140]
	Lysozyme and α-chymotrypsin	Poly (lactic-*co*-glycolic) acid	High encapsulation efficiencies (>70%) and residual activity (>90%).	[154]
Coacervation complex	Lysozyme	Poly (acrylic acid)-*block*-poly(acrylamide) Poly(*N*,*N*-dimethylaminoethyl methacrylate)	The stability of the micelles containing a larger fraction of lysozyme was lower.	[155]
Extrusion	α-amylase	Gelatin and shellac	The enzyme showed good stability after encapsulation and can be recycled 10 times.	[132]
Thermal gelation	l-phenylalanine ammonia-lyase	Plant hydrocolloids	Good results were obtained; however, new studies are necessary.	[156]
Spray drying	β-galactosidase	Chitosan	Encapsulation increased the diffusional effect of the released enzyme and reduced the initial activity of the enzyme.	[157]
	DNase I	Poly (lactic-*co*-glycolic) acid	High encapsulation efficiency (>80%); microparticles loaded with DNase I showed high inhalation rates and increased mucolytic activity.	[16]
Double emulsion	Laccase	Eudragitfi L 100-55	Increased stability of the enzyme at acidic pHs (2.0–5.0).	[158]
	α-chymotrypsin	Poly (ethylene glycol)-co-poly (glycerol adipate-*co*-ɷ-pentadecalactone)	Good throughput and encapsulation efficiency; encapsulation kept the bioactivity of α-chymotrypsin and protected it from adverse preparation conditions.	[159]
Layer-by-layer	Catalase	Poly (allylamine hydrochloride) dextran sulfate	Catalase remained active inside the polymer capsules; polymer capsules showed potential to prevent oxidative stress.	[160]
	l-asparaginase	Poly dextran/poly-l-arginine	Encapsulation improved proteolytic resistance and thermal inactivation of l-asparaginase.	[161]
Ionic gelation	Human phenylalanine hydroxylase	Chitosan	Effective in maintaining protein stability and enzymatic function.	[162]
	Bromelain	Chitosan	High encapsulation efficiency (85.1%); improved the stability of bromelain.	[138]
	Pectinase	Sodium alginate	Pectinase can be used to hydrolyze pectic substances in orange juice; maintenance of enzyme stability activity during recycles.	[163]
	Lipase	Sodium alginate and Chitosan	High encapsulation yield (99.8%); improvement of enzyme activity.	[57]

**Table 2:** Application of polymer encapsulated enzymes.

Enzyme	Method	Application	Refs.
Lysozyme	Electrospinning	Drug delivery/delivery of biopharmaceuticals to the oral mucosa.	[19]
β-galactosidase	Electrospinning	Oral drug delivery.	[152]
Fructosyltransferase	Electrospinning	Biocatalysts.	[141]
Papain	Electrospinning	Wound debridement.	[190]
Phosphatase	Freeze-drying	Reaction engineering.	[17]
Bromelain	Freeze-drying/ionic gelation	Wound healing and blood circulation improvement.	[138]
l-asparaginase	Ionic gelation	Drug release.	[167]
Pectinase	Ionic gelation	Clarifying orange juice.	[163]
Flavourzyme	Ionic gelation	Cheese ripening.	[14]
Aminopeptidase	Ionic gelation	Food industry: accelerating; cheddar cheese ripening through peptide hydrolysis.	[191]
Lysozyme; α-chymotrypsin	Nanoprecipitation	Novel treatments in immunology, oncology, or enzyme therapies.	[154]
DNAse 1	Spray-drying	Delivery of particulates carrying therapeutics to patients with cystic fibrosis.	[16]
